# Extracellular microRNAs and oxidative stress in liver injury: a systematic mini review

**DOI:** 10.3164/jcbn.17-123

**Published:** 2018-04-11

**Authors:** Juntaro Matsuzaki, Takahiro Ochiya

**Affiliations:** 1 Division of Molecular and Cellular Medicine, National Cancer Center Research Institute, 5-1-1 Tsukiji, Chuo-ku, Tokyo 104-0045, Japan

**Keywords:** microRNA, extracellular vesicle, liquid biopsy, liver injury

## Abstract

Recent evidence has suggested that extracellular microRNAs have crucial roles in intercellular communications and are promising as minimally invasive biomarkers for various diseases including cancers. Oxidative stress also plays an essential role in homeostasis and disease development. This systematic review aims to clarify the current evidence on the interaction between oxidative stress and extracellular microRNAs. We identified 32 studies that provided information regarding the association between oxidative stress and extracellular microRNAs: 9 focused on the central nervous system, 11 focused on cardiovascular diseases, and 4 focused on liver injury. Endothelial cell-specific miR-126-3p was the most studied extracellular miRNA associated with oxidative stress. In addition, we highlight some reports that describe the mechanisms of how oxidative stress affects extracellular microRNA profiles in liver injury. In liver injury, the levels of miR-122-5p, miR-192-5p, miR-223-3p, and miR-1224-5p were reported to be elevated in the sera. The release of miR-122-5p, miR-192-5p, and miR-1224-5p from hepatocytes may be attributed to oxidative stress. miR-223-3p could be released from neutrophils and suppress oxidative stress in the liver. Elucidation of the mechanisms of the interaction between extracellular microRNAs and oxidative stress would improve our pathophysiological understanding as well as future medical practice.

## Introduction

In multicellular organisms, cells can exchange information using single molecules such as hormones and cytokines. In addition, recent evidence has revealed that extracellular vesicles (EVs; also known as exosomes and microvesicles) can transport various molecules, such as nucleic acids, proteins, and lipids, between cells and play pathophysiological roles.^(1,2)^ MicroRNAs (miRNAs) are the most investigated EV components thus far.^(3–5)^ miRNAs are endogenous, short regulatory RNA molecules of 19–25 nucleotides in length. They modulate target gene expression at the post-translational level by guiding the RNA-induced silencing complex to miRNA target sites in the 3' untranslated region of mRNAs, leading to mRNA degradation or the inhibition of translation.^(6)^ Currently, 2,588 mature human miRNAs are listed in the miRNA registry (miRBase release 21^(7)^). Among them, 300–500 miRNAs can be detected as stable extracellular miRNAs in circulation. In addition to those encapsulated within EVs, some extracellular miRNAs are bound to either RNA-binding proteins^(8)^ or high-density lipoproteins.^(9)^ As changes in extracellular miRNA profiles are associated with various disease conditions, they are attractive candidates for minimally invasive biomarkers.^(10)^ Furthermore, studies on extracellular miRNA have revealed functional cell-to-cell miRNA transfer.^(11,12)^

Oxidative stress resulting from the increased production or inadequate removal of reactive oxygen species (ROS) plays a key role in the pathogenesis of aging, atherosclerosis, Alzheimer’s disease, cancer, etc. As both extracellular miRNAs and oxidative stress are essential in homeostasis and disease development, knowledge about the interaction between them could provide new physiological insights. Here, we systematically reviewed the literature to clarify the current progress in the field and highlight some notable studies on digestive diseases, which can enhance our understanding of the field and point out future directions for investigation.

## Methods

We searched MEDLINE for studies published until 21 December 2017 using a search strategy (("oxidative stress"[MeSH Terms] OR ("oxidative"[All Fields] AND "stress"[All Fields]) OR "oxidative stress"[All Fields]) AND ("micrornas"[MeSH Terms] OR "micrornas"[All Fields] OR "microrna"[All Fields])) AND (circulating[All Fields] OR ("serum"[MeSH Terms] OR "serum"[All Fields]) OR ("plasma"[MeSH Terms] OR "plasma"[All Fields])) OR ("exosomes"[MeSH Terms] OR "exosomes"[All Fields] OR "exosome"[All Fields]) OR extracellular[All Fields] OR salivary[All Fields] OR ("urinary tract"[MeSH Terms] OR ("urinary"[All Fields] AND "tract"[All Fields]) OR "urinary tract"[All Fields] OR "urinary"[All Fields]) OR juice[All Fields] OR fluid[All Fields] OR ("ascites"[MeSH Terms] OR "ascites"[All Fields]) OR effusion[All Fields] OR ("feces"[MeSH Terms] OR "feces"[All Fields] OR "fecal"[All Fields])) NOT ("review"[Publication Type] OR "review literature as topic"[MeSH Terms] OR "review"[All Fields]).

We included English-written original articles that described the association between circulating miRNAs and oxidative stress in their assessment. The exclusion criteria employed are as follows: papers that were not written in English, review articles, papers that only concerned intracellular miRNAs, papers that mentioned the association between circulating miRNAs and oxidative stress only based on *in silico* prediction, and papers that did not focus on human health or diseases.

## Results

### Result of literature search

**Fig. 1:**
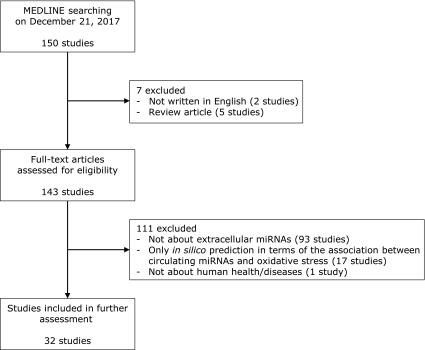
Workflow of the systematic review.

We obtained 150 published studies from the initial search, 32 of which were identified as focusing on the association between extracellular miRNAs and oxidative stress (Fig. 1). The organs/diseases in focus and reported miRNAs in these 32 studies are listed in Table 1.^(13–44)^ These were published after 2013, and the number of studies increased every year. More than half of the identified studies were focused on cardiovascular diseases and/or the central nervous system.

**Fig. 2:**
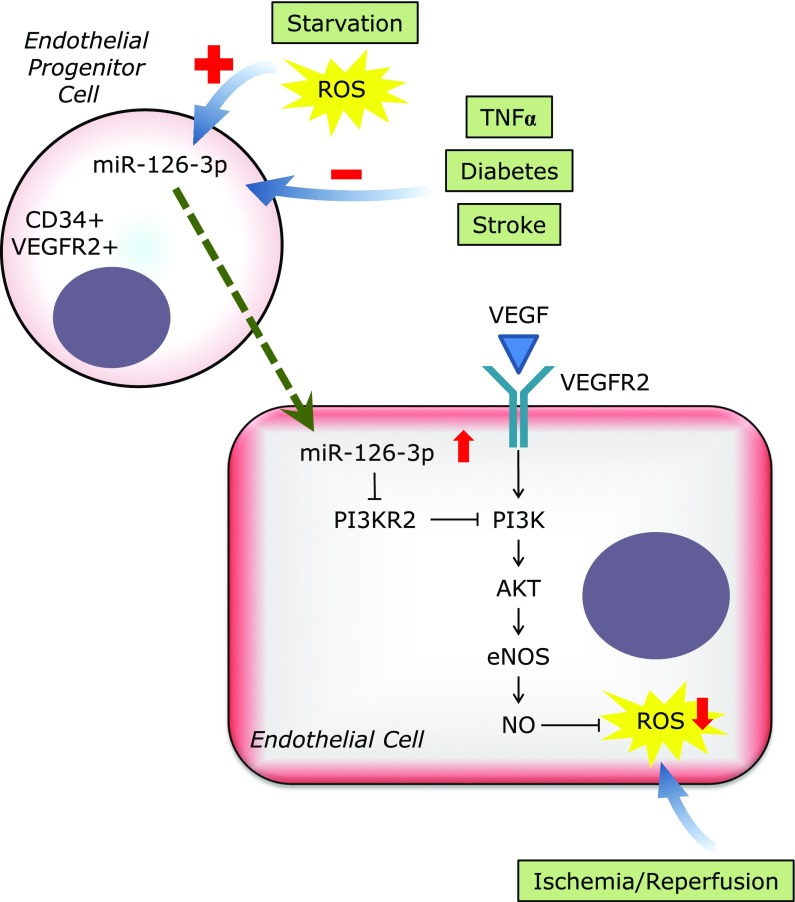
Function of endothelial progenitor cell-derived miR-126-3p in endothelial cells. ROS, reactive oxygen species.

Among these 32 studies, the most frequently documented miRNA was miR-126-3p. As miR-126-3p is located within intron 7 of the EGF-like domain 7 (Egfl7), which is an endothelial-cell-derived secreted peptide, miR-126-3p is specifically expressed in endothelial cells.^(45,46)^ In endothelial cells, miR-126-3p promotes angiogenesis by inhibiting endogenous VEGF repressors (SPRED1 and PIK3R2) (Fig. 2).^(47)^ In addition, miR-126-3p in cardiomyocytes has the function of suppressing cardiac inflammation, macrophage infiltration, oxidative stress, and fibrosis according to the data from endothelial cell-specific conditional miR-126 knockout mice.^(17)^ Circulating miR-126-3p is known to be suppressed in patients or animal models of cerebral ischemic stroke, diabetes, or peripheral arterial disease.^(17,24,27)^ Furthermore, oxidative stress might play a role in releasing circulating miR-126-3p as oral treatment with the antioxidant *N*-acetylcysteine (NAC) was found to prevent the maximal exercise-induced increase of circulating miR-126-3p in patients with intermittent claudication.^(24)^ Wang *et al.*^(23)^ reported that miR-126-3p contained in endothelial progenitor cell-derived microvesicles (EPC-MVs) can suppress oxidative stress and promote angiogenesis of endothelial cells via the PI3K/eNOS/NO pathway. They also showed that miR-126-3p was increased in MVs released from EPCs cultured in a serum deprived medium (starvation stress), whereas it was decreased in MVs released from EPCs cultured in a serum-deprived medium containing tumor necrosis factor-α (TNFα) (apoptotic stress). This example clearly showed that oxidative stress can be a regulator of extracellular miRNAs, and can be regulated by extracellular miRNAs.

### Identified studies about liver injury

**Fig. 3:**
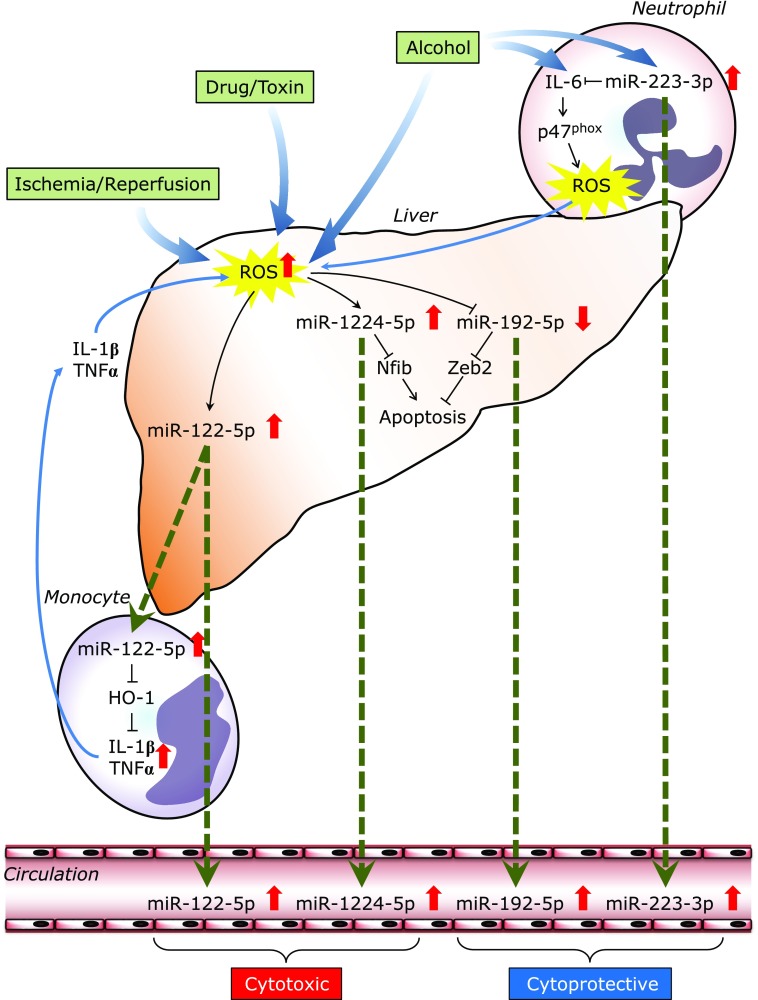
Regulatory mechanisms of circulating/extracellular miRNAs in liver injury. ROS, reactive oxygen species.

Regarding the digestive system, 4 studies were focused on liver injury as indicated in Table 1. Here, we introduced miRNAs that are associated with oxidative stress and released from cells during liver injury (Fig. 3). Nevertheless, the findings could be applied to all pathophysiological events in which ROS-mediated extracellular miRNAs are involved.

miR-122-5p is one of the most investigated miRNAs; it is abundantly expressed in hepatocytes but is absent or expressed at very low levels in other cell types.^(48)^ Mice lacking the gene encoding miR-122-5p are viable but develop temporally controlled steatohepatitis, fibrosis, and hepatocellular carcinoma.^(49)^ In healthy individuals, the number of exosomes containing miRNA-122-5p significantly increases in the serum after alcohol binge drinking.^(50)^ Exosomes derived from ethanol-treated human hepatoma cells (Huh7.5 cells) are taken up by monocytes/macrophages and Kupffer cells and horizontally transfer miR-122-5p. In monocytes, exosome-transferred miR-122-5p inhibits the HO-1 pathway and increases the levels of pro-inflammatory cytokines such as IL-1β and TNFα. Mosedale *et al.*^(44)^ reported that early increases in exosomal miR-122-5p tend to be associated with mitochondrial-induced apoptosis and oxidative stress during idiosyncratic drug-induced liver injury. Taken together, alcohol- or drug-induced cellular stress would promote the release of miR-122-5p-containing exosomes in hepatocytes in the absence of overt necrosis. Circulating miR-122-5p could be an early biomarker for damaged hepatocytes.

Although miR-1224-5p is not a liver-specific miRNA, Roy *et al.*^(35)^ discovered that it is also up-regulated in the serum of patients with acute liver failure. Intracellular miR-1224-5p was found to be up-regulated in hepatocytes following *in vivo* and *in vitro* ischemia-reperfusion or H_2_O_2_ stimulation. They also demonstrated that miR-1224-5p could suppress the anti-apoptotic gene Nfib, leading to impaired proliferation and elevated apoptosis. More importantly, increased serum levels of miR-1224-5p were found to be associated with survival in acute liver failure (area under a receiver operating characteristic curve, 0.92).

The same researchers also reported that serum miR-192-5p levels are selectively elevated in patients with liver injury and closely correlated with serum miR-122-5p levels.^(34)^ Supernatant levels of miR-192-5p were also found to be increased in a hypoxia/reoxygenation model of *in vitro* hepatocyte injury. However, in contrast to the up-regulation of miR-122-5p and miR-1224-5p in hepatocytes, miR-192-5p was reported to be down-regulated in injured livers *in vivo* and during H_2_O_2_ stimulation *in vitro*. Functional experiments confirmed the protective effect of miR-192-5p down-regulation in hepatocytes through the increase of a target gene (Zeb2), an important suppressor of apoptosis. Based on these results, the authors suggested that the decrease in intracellular miR-192-5p could be caused by the release of miR-192-5p from hepatocytes during acute liver injury. A limited number of reports also show reciprocal changes in intracellular and extracellular miRNAs, suggesting that some miRNAs might be actively and selectively released from cells in specific conditions.^(51,52)^

Li *et al.*^(22)^ found that the serum miR-223-3p levels of alcoholics were elevated compared with those of healthy controls by miRNA microarray analysis, and miR-223-3p could also be a possible biomarker for alcoholic liver injury. However, miR-223-3p was not released from hepatocytes but present at high levels in neutrophils. In mice, the levels of miR-223-3p were found to be increased in both the serum and neutrophils upon ethanol intake. They also showed that miR-223-3p could directly inhibit IL-6 expression and subsequently inhibit p47^phox^ expression in neutrophils. In miR-223-3p-deleted mice, the expression of IL-6 and the phagocytic oxidase p47^phox^ was enhanced in the liver, leading to ROS generation, neutrophil infiltration, and hepatic injury upon ethanol administration. ROS production by neutrophils and ethanol-induced liver injury were suppressed by p47^phox^ deletion. In summary, miR-223-3p in neutrophils could be an important regulator for blocking neutrophil infiltration in alcoholic liver disease.

## Discussion

In this systematic review, we identified 23 studies indicating that oxidative stress could affect extracellular miRNA profiles and that some transported miRNAs could play cytotoxic or cytoprotective roles in recipient cells. Although a number of studies addressed the use of extracellular miRNAs as biomarkers for various diseases, the regulatory mechanisms of extracellular miRNAs remain unclear. Further studies on oxidative stress should be conducted to shed light on this issue.

In the case of digestive diseases, all studies on the association between oxidative stress and extracellular miRNAs were focused on liver injury. In acute liver injury and hepatitis, circulating miRNAs regulated by intrahepatic oxidative stress seem to be powerful assessment tools for determining the extent of liver damage. The most important requirement for the use of a biomarker of acute diseases, such as acute liver injury, acute pancreatitis, and acute myocardial infarction, is to facilitate rapid measurement. Since it takes 1 to 3 days to obtain results by conventional quantitative RT-PCR or microarray, novel methods are required to use circulating miRNAs for the evaluation of acute diseases.

In digestive carcinogenesis, exposure to oxidative stress plays crucial roles.^(53)^ In addition, hundreds of previous reports have shown that circulating miRNA signatures are dramatically altered through the carcinogenic process.^(10,54)^ Nevertheless, none of the studies demonstrate the importance of the association between oxidative stress and extracellular miRNAs in cancer development or progression. As the aberrant miRNA profiles in cancer tissues and their precursor lesions such as Barrett’s esophagus, gastric intestinal metaplasia, or the inflamed colonic mucosa in ulcerative colitis are well-known,^(55–57)^ extracellular miRNAs released from malignant and pre-malignant lesions should be studied in detail with a focus on oxidative stress to promote cancer prevention and early detection.

In conclusion, although there are some well-conducted studies, knowledge of the association between oxidative stress and extracellular miRNAs is rapidly increasing but still limited. Further studies in this area would uncover unique cell-cell interactions and lead to changes in future clinical practice.

## Figures and Tables

**Table 1:** Oxidative-stress associated extracellular miRNAs

Focused organs/diseases	miRNAs	References
Central nervous system/Stroke	miR-4639-3p, miR-1-3p, miR-17-5p, miR-20a-5p, miR-20b-5p, miR-99a-5p, miR-106b-5p, miR-126-3p, miR-129-5p, miR-181c-5p, miR-300-3p, miR-369-3p, miR-410-3p, miR-451a, miR-499a-5p, miR-219a-5p	(Pusic, 2014; Pusic, 2015; Tao, 2015; Ma, 2016; Pusic, 2016; Chen, 2017a; Chen, 2017b; Li, 2017b; Phillips, 2017)
Cardiovascular diseases	miR-92a-3p, miR-126-3p, miR-129-5p, miR-155-5p, miR-210-3p, miR-21-5p, miR-29a-3p, miR-17-3p	(Wang, 2013; Curti, 2014; Chen, 2015; da Silva, 2015; Wang, 2015; Yamaguchi, 2015; DuPont, 2016; Wu, 2016; Xiao, 2016; Liu, 2017; Ramachandran, 2017)
Liver	miR-122-5p, miR-192-5p, miR-223-3p, miR-1224-5p	(Roy, 2016; Li, 2017a; Mosedale, 2017; Roy, 2017)
Kidney	miR-15a-5p, miR-92a-3p	(Kamalden, 2017; Shang, 2017)
Peripheral blood, bone marrow	miR-96-5p, miR-182-5p, miR-183-5p, miR-17-3p	(Curti, 2014; Davis, 2017)
Sepsis	miR-1-3p, miR-25-3p	(Yao, 2015; Xu, 2017)
Diabetes	miR-126-3p	(Wu, 2016)
Lung	miR-320a, miR-221-3p	(Lee, 2016)
Hyperlipidemia	miR-33a-5p, miR-33b-5p, miR-200c-3p	(D'Agostino, 2017)
Skin/Vitiligo	miR-25-3p	(Shi, 2016)
